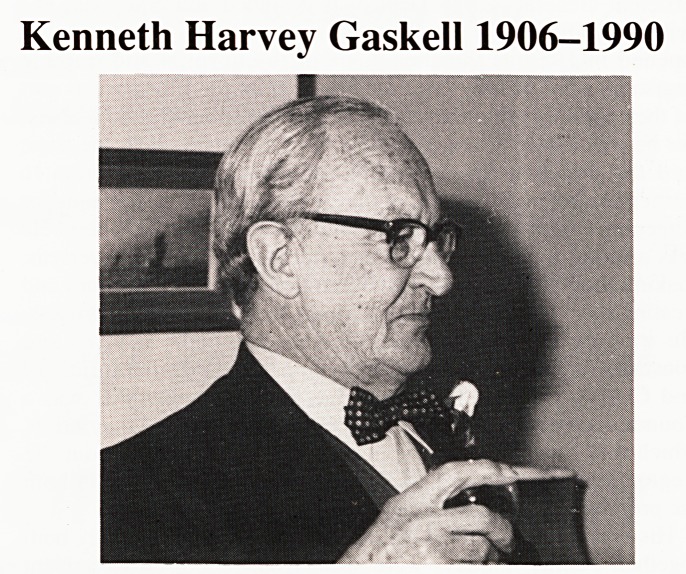# Dr. Kenneth Harvey Gaskell

**Published:** 1991-03

**Authors:** 


### Kenneth Harvey Gaskell 1906–1990

Kenneth Harvey Gaskell was born in 1906, the son of a doctor in Stretchford, in a house that had been designed by him to have no right angle corners, either between walls, or at the junctions of walls, ceilings and floors. The object was avoid the collection of dust, but the result was that the furniture had to stand away from the walls much to the irritation of his mother, nee Edith Mary Harvey, the last direct descendant of William Harvey of blood circulation fame. The father, in pursuit of medical history, had sought her out, for information, an expedition which had ended happily in marriage.

'Mac' Gaskell as he was later to be known, was educated at Repton, Cambridge and King's College Hospital, qualifying in 1931. His early house jobs in Orthopaedics to Sir Thomas

Fairbank and in Anaesthetics followed by a surgical registrar post at Hillingdale Hospital indicated an early desire to be involved on the practical side of medicine and this was apparent in his later career as a radiologist, where he excelled in examinations of the abdomen, and in the diagnosis of difficult orthopaedic problems. From 1933–38 he was in general practice in Woodchurch in Kent, with a appointment for X-Ray sessions at Ashford Hospital, where he had his own portable X-Ray machine, generator and dark room, and it was there that he developed an interest in Radiology. this took him back to Addenbrooke's Hospital to attend the 1938 D.M.R.E. training course, which was cut short by the war, for in 1939 he moved his family to Budleigh Salterton to be near his father, and then volunteered for war service. At first engaged in general duties, he was later posted as graded radiologist to Kaduna and Ibadan in 1942–3. Later he returned to Europe, serving in the Field Hospitals during the advance into Germany.

On demobilisation, after further study in London, he acquired his D.M.R.D., and then moved to Bristol with appointments in 1946 as assistant to Dr. J.V. Sparks at Southmead Hospital and Frenchay Hospital. Later he was Deputy Director at the Bristol Royal Infirmary, followed by a full-time appointment in Radiology at Frenchay and Cossham Hospitals in 1951, until his retirement in 1971.

Frenchay Hospital was at that time emerging from its role as a war-time hutted hospital, when it was used by the Americans. The buildings were primitive with brick walls and coke stoves placed centrally in the wards. With the advent of the N.H.S. staff were being appointed, and by the time that Mac arrived on the scene, it had become a Regional Centre for Thoracic Surgery, Plastic Surgery and Neuro Surgery. A handful of distinguished names would have welcomed him, such as Ronnie Belsey, Tom Wilton, Geoffrey Fitzgibbon, Denis Bodenham, George Alexander, Douglas Phillips and John Sparks, all appointed at much the same time in the 40s. The idea in 1950 was to create a General Hospital alongside the Regional Centres, and Mac was clearly to be the guiding hand in the Radiology Department. An empty ward was acquired and with the benefit of his Army training, a practical and efficient General X-Ray Dept. was designed and equipped; and with the addition of a few 'blisters' since, it is still in use today. It opened in 1954 as the showpiece of the hospital and training registrars were quickly integrated there from the Infirmary as part of their training programme. Mac was a particularly good teacher, for he was happy to spend endless time discussing both the basic and the difficult cases with them and encouraged them to participate in the active side of the Department where demands for screening examinations were quickly increasing in numbers. He was a father figure to many young doctors, some from overseas, making them feel at home, genial, modest, patient at all times and treating them as equals, making lasting friendships in the process. He was always keen himself to attend outside lectures and constantly encouraged others to accompany him to the Infirmary and Canynge Hall to attend routine lectures there. Mac was a Founder member of the South-West Radiologists Association, which met four times a year in different centres throughout the Region and was an early Chairman, participating keenly in both the professional side and the social side.

His great loves included his family and the sea, preferably both together. Family holidays were spent from school on an ancient ketch called Kelpie, which was kept at Parkstone in Poole Harbour, he being a member of the local Yacht Club. He first purchased a cottage at Salcombe in 1960. Five years later he purchased a larger house at Salcombe and exchanged his house in Clifton for a flat, thus allowing him to spend most weekends by the sea. He became a member of the Salcombe Yacht club, and owned a series of small sailing boats and motor/sailors until for the last few years he had a 20ft. diesel engined fishing boat with a cabin in which he cruised and fished in the Salcombe Estuary and along the South Devon coast. It was in the Estuary in 1981, fortunately whilst close to land, that he developed a dramatic illness which he himself diagnosed, correctly, as an incipient rupture of an abdominal aortic aneurysm. He managed to hail a passing ambulance which took him to the Vascular Unit in Plymouth where a trouser graft was successfully inserted. Truly a master clinician – no time for radiology in a crisis!

Another great love was motoring and the motor car. The fascinating story is told by his son Sir Richard, in the following words — "The interest started when he was at prep school and cycled to visit a Gaskell relative who lived at Fordsham in Cheshire. There they saw his two White steam cars, one was a black town Landau, a closed vehicle and other was an open four seater with off-white woven basketwork body and no hood. The Chauffeur took them for a ride, having first raised steam pressure. By 1920 at the age of 14, he had a Clyno two stroke motorbike and licence to ride it. At 16 he had a Raleigh two and three-quarter horse power four stroke motor bike. Father's first car was a Salmson 8 horse power cycle car, an open two seater made in 19212 which he had in 1923 until 1927 while he was at Cambridge. That was followed from 1927 to 1930 by an Austin 7 Gordon England Special 2 seater. This is the car which his cousin, who was nursing at King's College Hospital when he was there, told me that he used to drive down the corridors of the Hospital on odd occasions. between that car and his final one, the VW Golf Automatic, he had 23 assorted cars, much of the servicing of which he did personally until his last days. He drove until two months of his death, but in the last five years of his life covered less than 2,000 miles."

His modest self-written Obituary in the British Medical Journal of December 8 1990 concludes "Mac Gaskell is survived by his wife, Winsome; his son Richard; his daughter, Virginia (another daughter predeceased him); and six grandchildren."

J.L.G.T.

## Figures and Tables

**Figure f1:**